# Thermal Ablation and High-Resolution Imaging Using a Back-to-Back (BTB) Dual-Mode Ultrasonic Transducer: In Vivo Results

**DOI:** 10.3390/s21051580

**Published:** 2021-02-24

**Authors:** Hae Gyun Lim, Hyunhee Kim, Kyungmin Kim, Jeongwoo Park, Yeonggeun Kim, Jinhee Yoo, Dasom Heo, Jinhwan Baik, Sung-Min Park, Hyung Ham Kim

**Affiliations:** 1 Department of Biomedical Engineering, Pukyong National University, Busan 48513, Korea; haegyun@postech.ac.kr; 2 School of Interdisciplinary Bioscience and Bioengineering, Pohang University of Science and Technology, Pohang 37673, Korea; rlagusgml036@postech.ac.kr (H.K.); jpark215@postech.ac.kr (J.P.); jinhee.yoo@postech.ac.kr (J.Y.); 3 Department of Convergence IT Engineering, Pohang University of Science and Technology, Pohang 37673, Korea; kyungmin10@postech.ac.kr (K.K.); gun9509@postech.ac.kr (Y.K.); jinhwan52@postech.ac.kr (J.B.); sungminpark@postech.ac.kr (S.-M.P.); 4 Department of Mechanical Engineering, Pohang University of Science and Technology, Pohang 37673, Korea; hds9385@postech.ac.kr; 5 Department of Electrical Engineering, Pohang University of Science and Technology, Pohang 37673, Korea

**Keywords:** back-to-back structure, dual-mode transducer, high-frequency ultrasonic imaging, high intensity focused ultrasound (HIFU), high-resolution imaging

## Abstract

We present a back-to-back (BTB) structured, dual-mode ultrasonic device that incorporates a single-element 5.3 MHz transducer for high-intensity focused ultrasound (HIFU) treatment and a single-element 20.0 MHz transducer for high-resolution ultrasound imaging. Ultrasound image-guided surgical systems have been developed for lesion monitoring to ensure that ultrasonic treatment is correctly administered at the right locations. In this study, we developed a dual-element transducer composed of two elements that share the same housing but work independently with a BTB structure, enabling a mode change between therapy and imaging via 180-degree mechanical rotation. The optic fibers were embedded in the HIFU focal region of ex vivo chicken breasts and the temperature change was measured. Images were obtained in vivo mice before and after treatment and compared to identify the treated region. We successfully acquired B-mode and C-scan images that display the hyperechoic region indicating coagulation necrosis in the HIFU-treated volume up to a depth of 10 mm. The compact BTB dual-mode ultrasonic transducer may be used for subcutaneous thermal ablation and monitoring, minimally invasive surgery, and other clinical applications, all with ultrasound only.

## 1. Introduction

As an alternative to open surgery, a variety of minimally invasive cancer therapies using microwaves, radiofrequency, cryotherapy, or lasers have been developed. These techniques have been adopted widely because they can achieve the same or similar levels of treatment while reducing acute and chronic side effects such as pain, fatigue, poor appetite, and slow recovery [1,2,3,4]. Microwave tumor ablation works by heating the affected tissue with an applied electromagnetic field. Although the technique is widely used in clinical treatment, the microwaves still need to be delivered into the ablation zone for heating [5,6]. Unlike microwave ablation, radiofrequency tumor ablation uses an electric current-based technique that provokes thermal agitation around the device, whereas cryotherapy applies a freeze–thaw protocol that is associated with ice-ball formation (–20 °C to –40 °C) to remove malignant tissue. However, both modalities have some drawbacks—a prolonged treatment time (10–15 min between each treatment) and multiple overlapping ablations to create an adequate safety margin [7]. Laser thermal ablation (LTA) uses a flexible, bare fiber tip to deliver light energy that brings coagulative necrosis within seconds, but this technique is only applicable to melanoma due to a lack of penetration depth [8]. In addition, all of these heat-based, minimally invasive modalities require the operator to place instruments at precisely the right positions to remove the targeted tissue, and can inadvertently damage other blood vessels or organs because of the heat propagating through the device [9,10]. 

High-intensity focused ultrasound (HIFU) is an emerging technique for the treatment of lesions in tumorous diseases, neurological disorders, and cosmetic treatments for skin due to its non-invasive nature [11,12,13,14]. HIFU causes a rapid temperature increase in the target lesion, which results in coagulation necrosis [15,16], and has significant advantages, including cost-effectiveness, the ability to create label-free images, deep penetration depth, minimal invasiveness, shorter treatment time, and good feasibility of treatment in complicated locations [1,17,18,19]. To guide treatment, magnetic resonance (MR)-guided HIFU [20], computed tomography (CT)-guided HIFU [21], and ultrasound-guided HIFU [22] have been suggested to improve the efficacy and safety of HIFU therapy and satisfy the needs of clinicians. MR-guided HIFU has a clear advantage because it allows for mapping of the local temperature distribution around lesions by providing dynamic temperature-sensitive images with high spatial resolution [20]. However, because this imaging modality is performed in quasi-real-time, it is difficult to examine and track changes that occur during treatment, especially while the MR thermometry zone is moving. The CT-guided HIFU system also enables imaging and therapy simultaneously with a contrast-enhanced CT agent, which allows monitoring of thermal ablation with HIFU [21]. When there is a concern over contrast agent use, CT contrast agents should be injected [23]. 

Given the advantages of ultrasound imaging, including easy accessibility, label-free imaging, and safety, a variety of ultrasound-guided therapies have been used clinically for spinal anesthesia and brachial plexus block (BPB) [24,25]. In particular, real-time ultrasound visualization of HIFU treatment has been well-developed over the last decade. A dual-frequency sensor based on a capacitive micromachined ultrasonic transducer (CMUT) combines imaging and HIFU systems into one integrated circuit [26]. Recently, integrated ultrasonic systems that combine imaging and HIFU with mechanical scanning in both the transverse and sagittal planes have been developed for the treatment of localized prostate cancer [27]. This “see and treat” technology is especially well applied in the skin treatment field, as it provides advantages in ultrasound imaging of the dermal and subcutaneous tissue layers down to 4.5 mm [28]. From this point of view, a commercialized device for skin lifting and tightening, Ultherapy (Merz Aesthetics, Raleigh, NC, USA), is widely used to allow clinicians to see the skin layers that they are treating [22]. However, the device suffers from a lack of power efficiency and image resolution because only one acoustic element is used for both imaging and treatment rather than an optimized design, which would refine the type of piezoelectrical material, center frequency, aperture size, etc. for each mode. Moreover, the coaxial design including single and ring transducers that radiates both modes through the same direction for ultrasonic propagation [28] or combines with other modality such as optical coherence elastography [29] results in (1) limited space for the imaging transducer (poor lateral resolution), (2) crosstalk between adjacent elements, and (3) a large footprint [28,29]. It should be noted that a single-element imaging transducer does not have a large enough aperture to generate acceptable spatial resolution, which results in the need for an electronic matching circuit or additional adjusting system [22,30]. 

In this study, for the first time, we developed a back-to-back (BTB) structured transducer for high-frequency ultrasound imaging and thermal treatment. This back-to-back structure employs two elements, one for HIFU therapy and the other for imaging, integrated on the opposite sides of a device. It offers an immediate evaluation of the HIFU treated area by obtaining high-resolution ultrasound images before and after the treatment. Previously available low-frequency (2 MHz to 6 MHz) imaging systems cannot clearly distinguish skin lesions [31,32,33], but an optimized BTB dual-mode transducer (composed of two elements that share the same housing but work independently) can provide more detailed imaging of superficial structures and irregular surfaces, including the dermis and upper layers of subcutaneous tissue in humans. Furthermore, since it is important to apply delicate and detailed procedures to subcutaneous layers during skin treatment, we increased the efficiency of the transducer by press-focusing and increasing the focal strength while reducing the aperture size of the transducer. 

In this paper, structural in vivo imaging (B-mode and C-scan) of live mice before and after thermal treatment was obtained with 20.0 MHz ultrasound with a lateral resolution of 200 μm. The temperature increase of the tissue caused by the therapeutic transducer was calibrated by the fiberoptic temperature measurement system. Comparison of images before and after treatment confirmed the significance of hyperechoic marks observed after HIFU ablation. The combination of two independent transducers for imaging and treatment in one BTB housing will have clinical applications in skin imaging and subsequent diagnosis and therapy.

## 2. Materials and Methods

### 2.1. Fabrication of BTB Dual-Mode Transducer 

**Figure 1:**
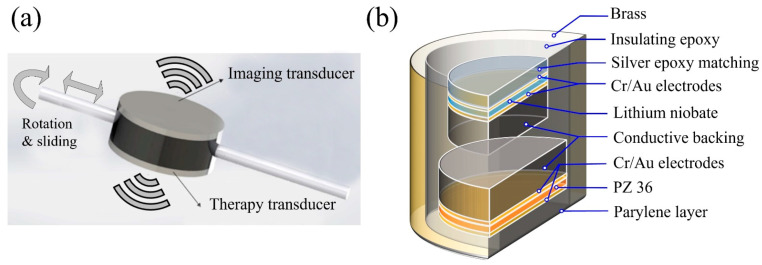
Back-to-back (BTB) dual-mode ultrasonic transducer for imaging and treatment; (**a**) the BTB structure was configured to have two modes—imaging and therapy; (**b**) internal construction of the BTB transducer.

To overcome previous design constraints (use of one transducer for both imaging and therapy or a limited amount of space available for the imaging transducer), a BTB structure was newly developed, as illustrated in Figure 1a. The fabrication process can be summarized into four parts, namely, (1) 20.0 MHz transducer for ultrasound imaging, (2) 5.3 MHz transducer for treatment, (3) BTB housing, and (4) assembly. The internal construction of the BTB transducer is shown in Figure 1b. The transducers were fabricated through the following procedure for creating a high-frequency transducer [34,35,36,37,38]. The optimized aperture size and thickness of the piezoelectrical material and the matching layer thickness for the individual imaging and therapeutic transducers were simulated with the Krimholtz, Leedom, and Matthaei models (PiezoCAD, Sonic Concepts, Bothell, WA, USA).

A 36° rotated Y-cut lithium niobate (LN) single crystal (Boston Piezo-Optics, Bellingham, MA, USA) was used for the 20.0 MHz imaging transducer, which was designed to have a large aperture size and low dielectric permittivity (ε_s_~39). It also offers high sensitivity due to its good electromechanical coupling coefficient (k_t_~0.49). Both sides of the lapped LN (157 μm) were sputtered with Cr/Au (500/1000 Å) electrodes using a sputtering machine. The first matching layer, a mixture of 2–3 μm silver particles (Silver, Aldrich Chemical Co., St. Louis, MO, USA) and Insulcast 501 epoxy (Insulcast 501, American Safety Technologies, Montgomeryville, PA, USA) was cured to the transmitting side of the LN and was lapped down to 10 μm. Conductive epoxy (E-solder 3022, Von Roll Isola, Schenectady, NY, USA) was cured to the bottom side of the LN. The entire acoustic stack was turned down to a diameter of 6 mm with a lathe. The surface of the LN was mechanically pressed to create a focal length of 13 mm.

Piezoceramic discs with a diameter of 8 mm (PZ36, Ferroperm Piezoceramics, KvistGaard, Denmark) were used in the 5.3 MHz treatment transducer due to their low acoustic impedance of 14 MRayl and high mechanical quality factor of 500. One side of the PZ36 was lapped and polished to a thickness of 337 μm to generate half-wavelength resonance. For the press-focusing process, a soft-silver epoxy-backed structure with a thickness of 500 μm was attached. Mechanical pressing of the PZ36 created a focal distance of 13 mm, which allowed the same focal spot as that of the 20.0 MHz transducer. A same-sized iron ball bearing was used for press-focusing.

Every acoustic stack for the 20.0 MHz and 5.3 MHz transducers was placed concentrically in a single BTB housing. Two stacks faced toward opposite directions and the gap between them was filled with insulating epoxy (Epo-tek 301, Epoxy Technology, Billerica, MA, USA). We sputtered the front surface of both stacks again to connect the electrical ground. As a final matching layer, a parylene film (12 μm) was coated onto the outside of the transducer using a parylene coating system (PDS 2010 Labcoter, SCS, Indianapolis, IN, USA). To successfully co-register the focal planes of the two transducers, the BTB housing and the stepper motor for rotation were designed in a computer-aided design process to align the two separate transducers on the same beam axis, as shown in Figure S1. In addition, as shown in Figure S2, the two acoustic stacks were aligned at the center of the housing. The rotation of the BTB transducer was another possible source of misalignment. The stepper motor was used to rotate the transducer by 180 degrees with an acceptable error, and a comparison of B-mode and C-scan images before and after HIFU treatment was made to confirm and compensate for any potential misalignment.

### 2.2. Transducer Performance Test

**Figure 2:**
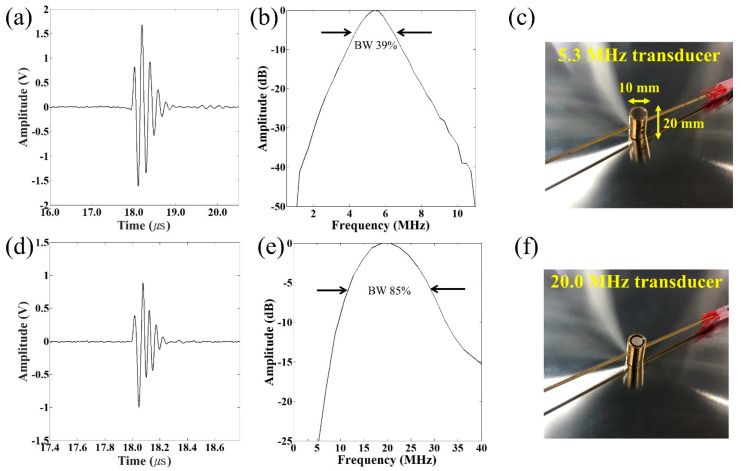
Pulse-echo response measurements and frequency spectra of 5.3 MHz and 20.0 MHz different-frequency transducers. (**a**) Echo response of a 5.3 MHz transducer; (**b**) frequency spectrum of a 5.3 MHz transducer; (**c**) photograph of the transducer of a 5.3 MHz transducer; (**d**) echo response of a 20.0 MHz transducer; (**e**) frequency spectrum of a 20.0 MHz transducer; and (**f**) photograph of the transducer of a 20.0 MHz transducer.

A pulse-echo response and the associated frequency spectra of two representative transducers at 5.3 MHz and 20.0 MHz were measured using a glass mirror as the imaging target. The pulser/receiver (DPR 500, JSR, Pittsford, NY, USA) was connected to the transducer and the transducer was excited by electrical impulses at a 500 Hz repetition rate with 50-Ω damping. Figure 2a,d shows the pulse-echoes of the transducers, and Figure 2b,e displays the frequency spectra. The HIFU transducer has a center frequency of 5.3 MHz and a −6 dB fractional bandwidth of 39%, while the corresponding numbers for the imaging transducer are 20.0 MHz and 85%. Figure 2c,f shows photographs of the 5.3 MHz and 20.0 MHz transducers, respectively. The frequency dependence of impedance and the phase angle of 5.3 MHz were measured with an impedance analyzer (E4990A, Keysight Technologies, Santa Rosa, CA, USA), as shown in Figure S3. 

**Figure 3:**
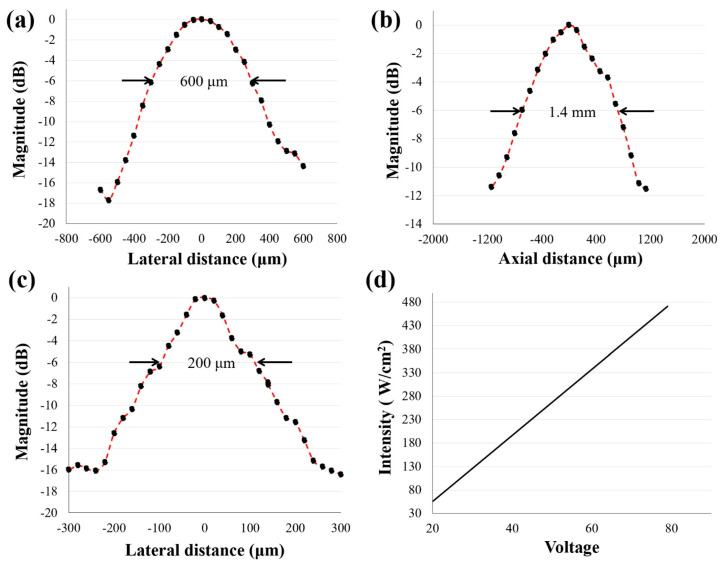
Acoustic pressure field of the BTB transducers measured with a needle hydrophone. (**a**,**c**) are one-dimensional lateral distributions for 5.3 MHz and 20.0 MHz transducers, respectively. The −6 dB lateral beam width was measured to be 600 μm and 200 μm, respectively; (**b**) depth of focus (DOF) of a 5.3 MHz transducer was measured and found to be 1.4 mm. Ultrasonic input parameters: Vpp = 20 V, cycle numbers of 3, and pulse repetition frequency of 1 kHz; (**d**) represents acoustic intensities at focus according to input voltages using a 5.3 MHz transducer.

A needle hydrophone (HGL-0085, Onda Corporation, Sunnyvale, CA, USA) was used to acquire lateral and axial pressure distributions (Figure 3a,b for the 5.3 MHz transducers and Figure 3c for the 20.0 MHz transducer). The driving conditions were as follows: driving frequency equal to each center frequency, input peak-to-peak voltage of 20 V, cycle number of 3, and pulse repetition frequency (PRF) of 1 kHz. The −6 dB lateral beam width was measured to be 600 μm and 200 μm for the 5.3 MHz and 20.0 MHz transducers, respectively. The −6 dB depth of focus (DOF) at 5.3 MHz was found to be 1.4 mm. Furthermore, the acoustic intensities versus corresponding input voltages at the transducer focal point are plotted in Figure 3d. 

### 2.3. Transducer Performance Simulation 

**Figure 4:**
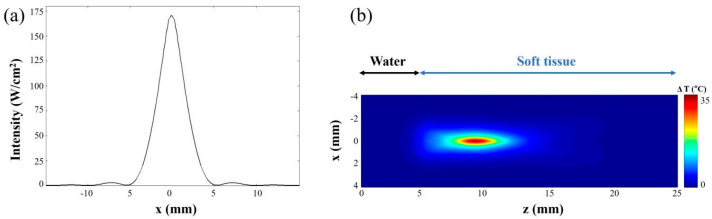
Simulated acoustic characteristics of the 5.3 MHz transducer when HIFU signal is applied. (**a**) The intensity of transducer at the focal point and (**b**) the temperature change region along the axial directions based on the linearized Khokhov–Zabolotskaya–Kuznetsov (KZK) wave equation and the bioheat transfer (BT) equation.

To evaluate the performance of the 5.3 MHz transducers for HIFU treatment, the HITU Simulator v2.0 (United States Food and Drug Administration, Silver Spring, MD, USA) was used [39]. The simulator predicts characteristics of nonlinear propagation of waves and high-intensity therapeutic ultrasound (HITU) beams, followed by heating effects. This is carried out by integrating Kokhov–Zabolotskaya–Kuznetsov (KZK) nonlinear wave equation [40] from the frequency-domain perspective to acquire power density Q, and the temperature change was calculated using the bioheat transfer (BT) equation, as follows:

(1) $${\rho C}_{p}\frac{\partial T}{\partial t}=\kappa \nabla^{2}T\;-\;wT\;+\;Q$$

where ρ (kg/m^3^) is the mass density, $C_{p}$ (J/kg°C) is the heat capacity, *T* (°C) is the temperature rise above equilibrium, κ (kg/m^3^s) is the thermal conductivity, and w (kg/m^3^s) is the blood perfusion rate [39]. In the simulation, the distance from the transducer to the target was set to 10 mm (5 mm of water and 5 mm of soft tissue). The detailed environment parameter values used for simulation are summarized in Table 1. Equation (1) was used in the simulation on the assumption that the power deposition resulting from metabolism was very small compared to the external power deposition [39]. Figure 4a shows the simulated intensity distribution at the focal spot. Figure 4b is the maximum temperature distribution at the focal area along the axial direction. The ultrasonic input parameters for the simulation are the same as those used for the in vivo experiments in mice. The results reveal that our HIFU input signal can generate enough power to increase the temperature by more than 35 °C. It is notable that the 10 °C -temperature increase did not generate any tissue necrosis at the focal area and was the safety threshold in the simulation. 

### 2.4. Ex Vivo Temperature Calibration

**Figure 5:**
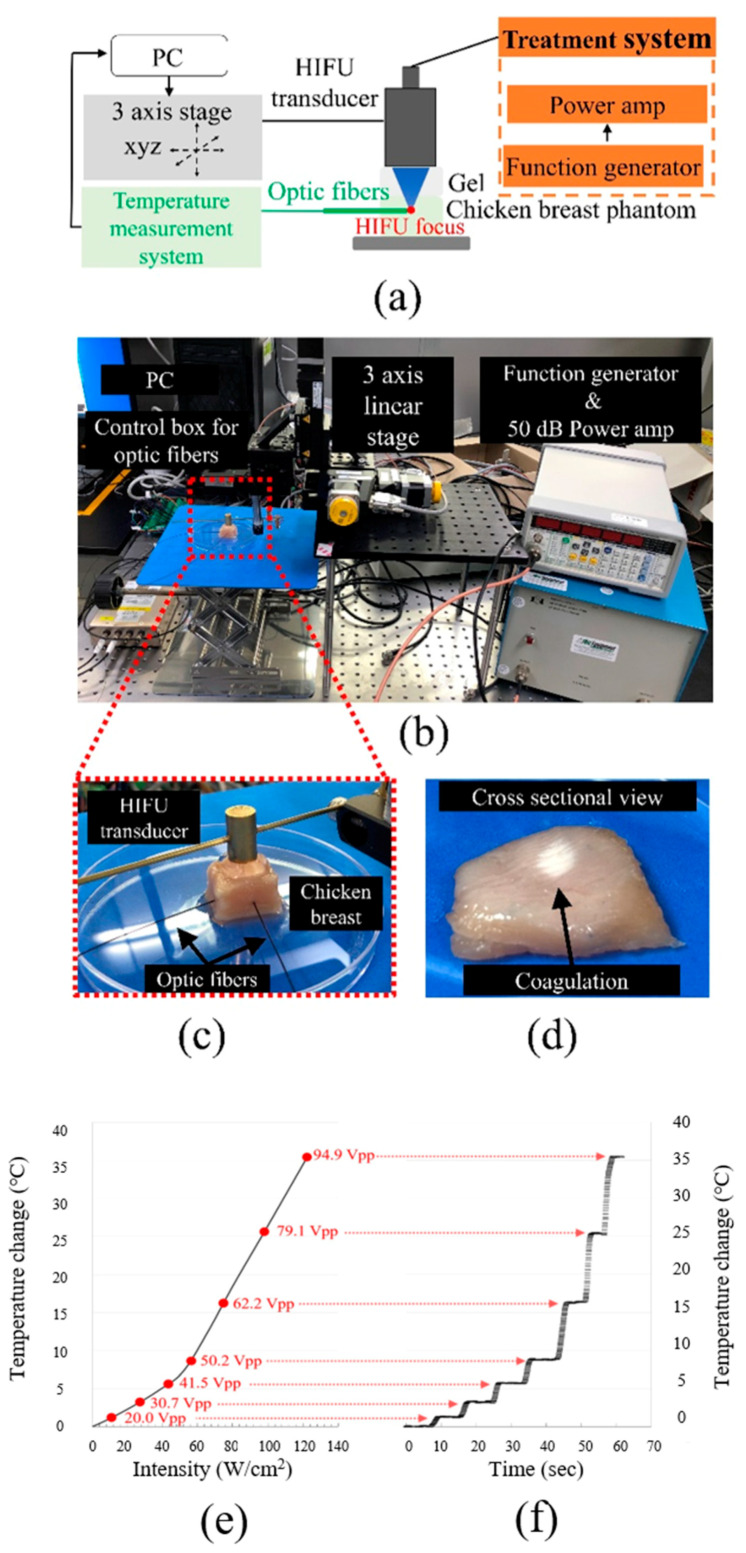
Temperature calibration using optic fibers. (**a**) Schematic illustration of temperature measurement; (**b**) experimental setup including two optic fibers and a control box; (**c**) two fibers were embedded in chicken breasts, and only the therapeutic transducer was operated to calibrate the thermal effects; (**d**) a cross-sectional view after HIFU treatment. Lesion obtained ex vivo from chicken by HIFU. Results of temperature calibration; (**e**) the temperature change over the intensity; and (**f**) the temperature change over time at given voltages.

Precise measurement of the temperature rise caused by various acoustic outputs was required to operate the designated HIFU treatment safely and effectively. Direct temperature measurements at the ultrasonic focal point of the 5.3 MHz transducer were taken during HIFU exposure in chicken breasts. As shown in the schematic diagram and experimental setup in Figure 5a–c, a fiberoptic thermometry system (Luxtron m600, Lumasense Technologies, Santa Clara, CA, USA) was used to measure the temperature distribution at the ultrasound focal area. Two fibers, temperature sensors, were embedded in chicken breasts at room temperature. A pulser-receiver was utilized to focus the transducer at the fiber tip. Figure 5d shows a cross-sectional photograph of lesions generated in the ex vivo chicken breast by cutting into a slice at the ultrasonic focal area with an average spatial peak pulse intensity (I_SPPA_) of 122.7 W/cm^2^. The white lesions are areas in which coagulation necrosis occurred due to treatment at the ultrasonic focus. Interestingly, no significant change in the color of the tissue was seen near the ultrasonic field. A direct comparison of the measured temperature change and ultrasonic intensity is displayed in Figure 5e. For more information on temperature calibration, Figure S4 demonstrates the temperature change over time at the given voltages and the rate of temperature increase. With a fixed PRF of 1 kHz and a cycle number of 250, various input voltages were applied, from 0 V_pp_ to 94.9 V_pp_. Direct temperature measurements confirm that the HIFU effect can be adjusted in certain areas of the tissue and that this adjustment depends on acoustic input parameters to provide vital guidance during thermal treatment procedures.

### 2.5. Phantom Imaging 

**Figure 6:**
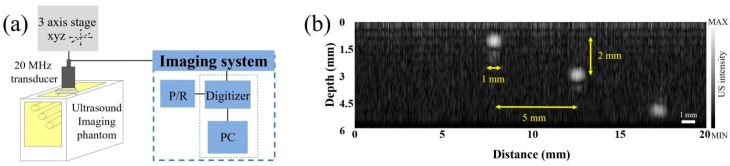
Phantom imaging for an evaluation imaging system using the 20.0 MHz transducer. (**a**) Schematic diagram of the ultrasound imaging system and (**b**) a phantom image with monofilament line targets with a diameter of 1 mm in the tissue-mimicking structure.

To characterize the performance of the 20.0 MHz imaging transducer, a commercial phantom (Model 539, ATS Laboratories, Bridgeport, CT, USA) was used. The imaging transducer was connected to an ultrasonic pulser-receiver (5073PR, Olympus NDT, Waltham, MA, USA) to obtain an echo signal. The pulser-receiver was manually set to a pulse repetition rate of 10 kHz, pulse energy of 16 uJ, and damping of 50 Ω, and was given 10 dB of receiving gain which drove a 180 V negative impulse to the transducer. A command signal from a customized LabView program was sent to a function data acquisition board (DAQ, PCIe-6320, National Instruments, Austin, TX, USA) that generated a signal to trigger the pulser-receiver. The DAQ initialized a digitizer board with a 200 MS/s sampling rate (PCI-5124, National Instruments, Austin, TX, USA) to acquire ultrasonic signals via the transducer, and the obtained signals were saved on a PC. The detailed schematic is shown in Figure 6a. Two-dimensional (2D) ultrasound images (B-mode) were achieved by mechanical scanning using x-axis motorized stages (L-509, Physik Instrumente, Karlsruhe, Germany). The field of view of the images was 20 mm × 6 mm, and the x-axis step sizes were 3.125 μm. The diameter of the target was 1 mm, and the lateral and axial displacements between the two targets were 5 mm and 2 mm, respectively, as shown in Figure 6b. The acquired ultrasound signals were Hilbert transformed and then median filtered to generate ultrasound images.

### 2.6. In Vivo Experiment: Imaging and Treatment Using BTB Transducer

**Figure 7:**
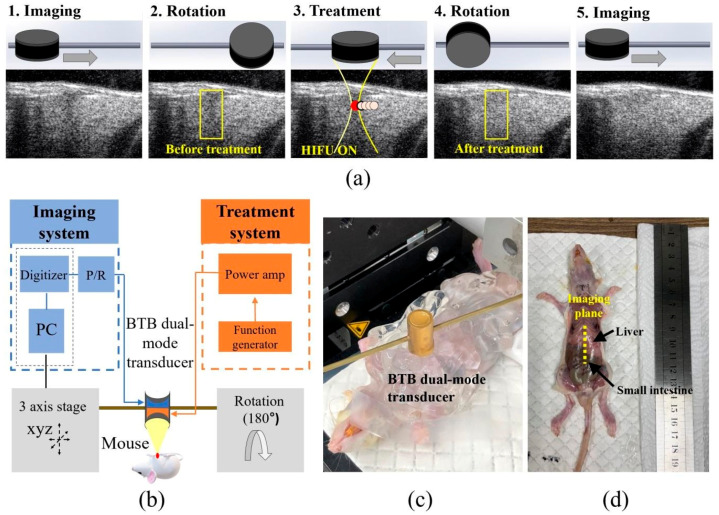
Ultrasound imaging and treatment using the BTB transducer. (**a**) Schematic illustration of the procedure used for the in vivo experiments: 1. imaging; 2. rotation; 3. treatment; 4. rotation; and 5. imaging; (**b**) the dual-mode system and its components; (**c**) a photograph taken during an in vivo experiment; and (**d**) mouse dissection after treatment.

All experimental procedures for the animal studies were conducted in accordance with the regulations of the Institutional Animal Care and Use Committee (IACUC) of Pohang University of Science and Technology (Approval number: POSTECH-2019-0077), and the regulations of the National Institutes of Health Guide for the Care and Use of Laboratory Animals. We used Balb/c mice (15 g, 6 weeks old) for the experiments (Figure 7c). The mice were anesthetized by injecting 3% vaporized isoflurane gas for 5 min in an air supply prior to the imaging and treatment session. The skin of each anesthetized mouse was depilated using commercially available hair removal cream (Veet, Reckitt Benckiser LLC, Parsippany-Troy Hills, NJ, USA). Ultrasound gel was applied to the skin of the experimental area as acoustic impedance matching media. The BTB transducer was connected to a pulser-receiver. Detailed information about the experimental setup and image processing is the same as that used for the phantom imaging described above, except for three-dimensional (3D) volumetric data collection and 180-degree rotation. Three-dimensional volumetric ultrasound images were achieved by mechanical scanning using two-axis x-y motorized stages (L-509, Physik Instrumente, Karlsruhe, Germany). The field of view of the images was 11 × 15 × 9 mm^3^ and the step sizes of the x- and y-axis were 25 μm and 30 μm, respectively. For the rotary stage, a stepper motor (PKP213D05A, Oriental Motor, Tokyo, Japan) rotated the BTB transducer in 100 increments (1.8 degrees each × 100 increments with 1 kHz pulse = 180 degrees). The total rotation time was about 0.1 sec. For the HIFU treatment, a PRF of 1 kHz, a cycle number of 250, and an input voltage of 94.9 V_pp_ were applied.

## 3. Results

The in vivo environment has different characteristics than the phantom environment; for example, temperature changes may differ according to the existing body temperature or heat generated by HIFU spreading into surrounding tissue due to blood flow. For validation purposes, live animal experiments were carried out using the finished BTB transducer, and the efficiency of the HIFU treatment was monitored with high-frequency ultrasonic imaging. 

The following basic principles and experimental steps of treatment and imaging with the BTB dual-mode ultrasonic transducer are illustrated in Figure 7a: (1) imaging before treatment; (2) 180° rotation; (3) treatment of the target region; (4) 180° rotation; and (5) final imaging to validate the treated region. For validation purposes, series of B- mode images were collected before and after treatment (Figure 7a). A schematic diagram of the in vivo imaging and therapy is shown in Figure 7b. The transducer was scanned from the mouse liver to the small intestine through the imaging plane noted in Figure 7d. Dissection was followed by HIFU treatment to confirm thermal damage on the outermost surface. Thermal ablation was not observed on the liver surface (Figure 7d). 

**Figure 8:**
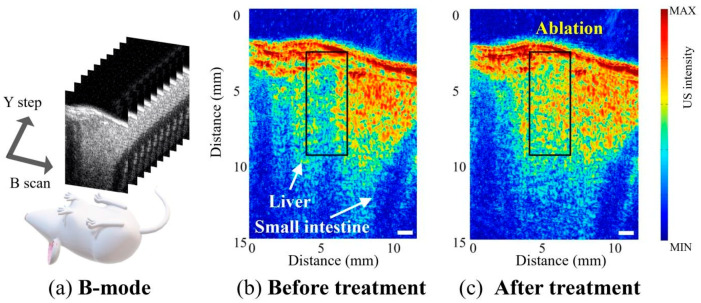
In vivo B-mode imaging. (**a**) Schematic of the B-mode imaging. Mouse bone and liver were imaged with high frequency ultrasound before (**b**) and after (**c**) treatment.

The color-scaled B-mode images (Figure 8b,c) acquired using the BTB transducer show the mouse liver and small intestine prior to and immediately following HIFU treatment. The liver lies at a depth of 2 mm below the skin. In general, tissue echogenicity is used as a means of monitoring tissue damage because any change in the hyperechoic properties in the image can be taken as a sign that the HIFU treated tissue has coagulated [41]. In this study, echogenic regions (ablation: rectangular box) were found in the B-mode images after treatment and therapy (Figure 8 and Supplementary Video S1). For HIFU treatments, it is very important to ensure that only the desired target volume becomes necrotic while neighboring regions are not damaged. As shown in Figure 8, the tissues around the target region were not adversely affected. High-frequency ultrasound imaging at 20.0 MHz with a lateral resolution of 200 μm is ideally suited for monitoring these echo-induced changes as these parameters overcome the resolution limitations of previous ultrasound-guided HIFU devices [25,26,27,28]. The use of a specular reflector along the small intestinal wall helps to emphasize the boundaries of the small intestine.

**Figure 9:**
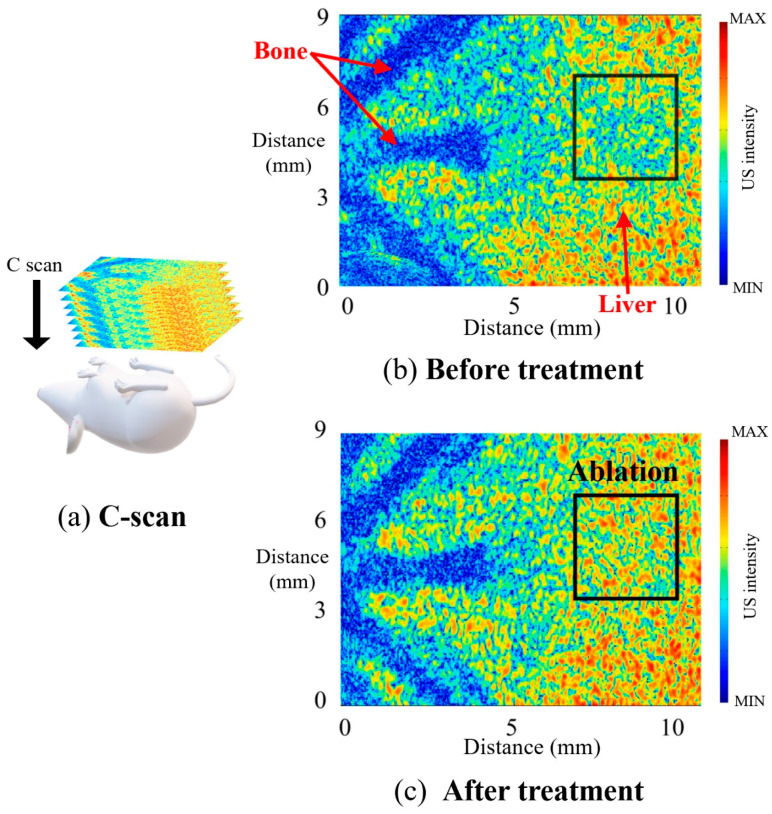
In vivo C-scan imaging. (**a**) Schematic of the C-scan imaging. Mouse bone and liver were imaged in high frequency ultrasound before (**b**) and after (**c**) treatment.

To localize the region of coagulation caused by HIFU treatment more accurately, C-scan 3D volumetric ultrasound images were obtained (Figure 9 and Supplementary Video S2). Hyper echogenicity at the site of HIFU lesion formation (ablation: rectangular box) is readily observable in Figure 9b,c. The echogenicity of the tissue is increased immediately after ablation. In this study, 2D B-scan and C-scan frames were collected and temporally visualized in video format to accurately detect the treated lesions (Supplementary Videos S1 and S2). Real-time 3D ultrasound facilitates clinicians’ diagnostic ability during surgery, and many studies conducted in recent decades have attempted to develop real-time ultrasound systems with dedicated traditional 2D array transducers. In further studies, post-imaging processing, including reconstruction and visualization for volume rendering, can be developed and applied to this system for real-time or near real-time 3D ultrasound imaging.

## 4. Discussion

The major goal of this paper is to introduce high-frequency ultrasound-guided HIFU treatment as a platform technology, which allows high-resolution visualization of the results of HIFU treatment in almost real-time. To the best of our knowledge, the current paper is the first to demonstrate the use of high-frequency in vivo imaging to monitor the dynamics of lesion formation using color-scale imaging after HIFU treatment. A new type of piezoceramic material with low acoustic impedance and high thickness coupling coefficient maximizes sensitivity, which leads to acceptable visualization of local necrosis within the therapeutic HIFU focus. Portability, low-cost, and fast imaging speed are the advantages of ultrasound over other imaging modalities such as magnetic resonance imaging (MRI) and CT. The use of high-frequency ultrasound (20.0 MHz) allowed high-resolution visualization of treated mouse liver tissue. Treated lesions were hyperechoic relative to pre-treatment lesions. This BTB imaging and therapy technique could offer highly reliable thermal cancer treatment, especially considering the small target size. Another advantage of focused transducers is the short duration of treatment (less than 10 s, which may be further reduced by optimization), making this thermal modality competitive with cryoablation and radiofrequency ablation [42,43,44]. Furthermore, this non-invasive and short-term treatment with dual-mode technology can be clinically applied to skin rejuvenation and tightening via heating of tissue to 65–70 °C, which causes collagen contraction, denaturation, and initiation of collagen synthesis [45,46]. 

Several alternatives have been developed for horizontal alignment of ultrasonic transducers, such as left-and-right, fore-and-aft, concentric dual, and back-to-back configuration, but their applications are limited in scope to imaging only [47,48]. Our approach, which uses a back-to-back structured transducer, provides a solution for both imaging and therapy and also solves the poor imaging quality problem common to previous configurations, which use a ring-shaped transducer for therapy and a circular transducer at the center for imaging. The limited available space for the imaging transducer (approximately 3 mm in diameter) did not provide sufficient lateral resolution due to its high f-number of ~5 to 6. However, the back-to-back configuration enables the use of a larger aperture for both the imaging (6 mm in diameter) and therapy transducers (10 mm in diameter) and therefore a low f-number of 2 to 3 for imaging, resulting in acceptable lateral resolution. 

To achieve both high-resolution imaging and power-efficient HIFU, ultrasound beams generated by two transducers should be focused using either a lens or a mechanically press-focused aperture. In general, lenses are not the preferred choice for high-frequency (greater than 15 MHz) ultrasound because lenses increase acoustic attenuation while transmitting and receiving signals. Moreover, HIFU does not usually employ a lens, which would act as a buffer layer between the piezoelectric material and the target tissue. Press-focusing is a technique to apply pressure to the acoustic stack and create a concave structure for focusing light as an alternative to a lens. As shown in Figure S2, two transducers were assembled on the opposite side of a single housing, with the same ball bearing. Therefore, both the imaging and therapy transducers have the same focal distance, which is required for co-registration of the HIFU-treated plane and high-resolution imaging plane when rotated by 180 degrees. 

Because the back-to-back transducer used in our study was for imaging and HIFU (not for imaging only, as in previous studies), two independent transducers were designed, one for imaging and the other for HIFU. The optimized size and thickness of each transducer were determined by the type of piezoelectrical materials (high-frequency imaging: lithium niobate, HIFU: PZ36 low acoustic impedance PZT) and their center frequencies (high-frequency imaging: 20 MHz, HIFU: 5.3 MHz in our case). Moreover, the f-number of each transducer was designed differently for their separate applications (high-frequency imaging: 2 to 3, HIFU: 1 to 2). 

The developed BTB transducer may be used for numerous applications that require high-resolution imaging and HIFU treatment in the superficial layer up to 10 mm. The first example is skin tightening treatment using HIFU. After treatment of the dermal layer located between the epidermis and subcutaneous muscle layer, the BTB transducer can offer high-resolution images of superficial structures for treatment verification [49]. Second, the BTB transducer may be used to ablate cutaneous melanomas that lie below the epidermal layer. Cutaneous melanoma has high rates of morbidity and mortality (65% of all skin cancer deaths). However, there is currently no ultrasound-guided HIFU technique for melanoma cancers [50]. Lastly, in small animal studies of human tumor models, tumor cells are injected between the muscle and skin layer for HIFU resection experiments. The effective volume of the incubated tumor should be at least 1100 mm^3^ at depths of 7–10 mm. The BTB transducer is suitable for high-resolution imaging and thermal therapy experiments for such incubated tumors [51].

## 5. Conclusions

In this study, we presented the development of a BTB dual-mode transducer for high-frequency imaging and HIFU treatment. Each mode of this dual-element transducer in a single housing is optimized, and the HIFU monitoring technique is improved. Optic fiber measurements were performed with temperature calibration generated by the HIFU transducer. In in vivo experimental studies, the capability of the dual-mode BTB transducer was verified with high-resolution images before and after treatment in a live animal environment at a focal distance below 5 mm from the skin, which is normally the subcutaneous layer in the human body. With implicit registration between the imaging and treatment axes, the reflected data could be processed, potentially in real-time, to visualize the therapeutic effect. The results illustrate the potential for near real-time feedback made possible by ultrasound imaging and will open the door for high-frequency ultrasound-guided HIFU. Possible applications of the BTB transducer include ultrasonic skin therapy and cancer treatment, especially in the superficial organ. Moreover, device development has progressed toward high-frequency array transducers with beam steering, which allows for a wider imaging area. Although the design of the device, including the transducer and supporting devices, may be complicated because of the many elements involved, the advantages gained for imaging would be significant.

## Figures and Tables

**Table 1:** Parameters used in the simulation.

Layer	Water	Soft Tissue
Sound speed (m/s)	1482	1629
Mass density ($\mathrm{kg}/m^{3}$)	1000	1000
Attenuation at 1 MHz (dB/m)	0.217	58
Fraction due to absorption	0.0	0.9
Exponent of attenuation power law	2.0	1.0
Nonlinear parameter	3.5	4.5
Heat capacity (J/kg/K)	4180	4180
Thermal conductivity (W/m/K)	0.6	0.6
Perfusion rate $(\mathrm{kg}/m^{3}$/s)	0.0	20

## Data Availability

Experiment data are available on request to Hyung Ham Kim.

## Supplementary Materials

The following are available online at https://www.mdpi.com/1424-8220/21/5/1580/s1, Figure S1: Computer-aided design for the back-to-back (BTB) transducer and the stepper motor for the rotation, Figure S2: Schematics of a press-focusing method for the BTB transducer, Figure S3: The electrical impedance and phase of the HIFU transducer measured by impedance analyzer, Video S1: Two-dimensional B-scan frame collection, Video S2: Two-dimensional C-scan frame collection.

## Patents

The authors have filed provisional Korea Patent 10-2019-0127026.

## References

[1] Wu F., Wang Z.B., Chen W.Z., Wang W., Gui Y., Zhang M., Zheng G., Zhou Y., Xu G., Li M. (2004). Extracorporeal high intensity focused ultrasound ablation in the treatment of 1038 patients with solid carcinomas in China: An overview. Ultrason. Sonochem..

[2] Eisele R.M., Denecke T., Glanemann M., Chopra S.S. (2014). Minimal-invasive microwave coagulation therapy for liver tumours: Laparoscopic and percutaneous access. Zentralbl. Chir..

[3] Li D., Kang J., Golas B.J., Yeung V.W., Madoff D.C. (2014). Minimally invasive local therapies for liver cancer. Cancer Biol. Med..

[4] Rozenblum N., Zeira E., Scaiewicz V., Bulvik B., Gourevitch S., Yotvat H., Galun E., Goldberg S.N. (2015). Oncogenesis: An “Off-Target” Effect of Radiofrequency Ablation. Radiology.

[5] Cavagnaro M., Amabile C., Bernardi P., Pisa S., Tosoratti N. (2011). A minimally invasive antenna for microwave ablation therapies: Design, performances, and experimental assessment. IEEE Trans. Biomed. Eng..

[6] Kim S.K., Rhim H., Kim Y.S., Koh B.H., Cho O.K., Seo H.S., Kim Y. (2005). Radiofrequency thermal ablation of hepatic tumors: Pitfalls and challenges. Abdom. Imaging.

[7] Hinshaw J.L., Littrup P.J., Durick N., Leung W., Lee F.T., Sampson L., Brace C.L. (2010). Optimizing the protocol for pulmonary cryoablation: A comparison of a dual- and triple-freeze protocol. Cardiovasc. Interv. Radiol..

[8] Choudhary S., Tang J., Elsaie M.L., Nouri K. (2011). Lasers in the treatment of nonmelanoma skin cancer. Dermatol. Surg..

[9] Sartori S., Di Vece F., Ermili F., Tombesi P. (2017). Laser ablation of liver tumors: An ancillary technique, or an alternative to radiofrequency and microwave?. World J. Radiol..

[10] Pillai K., Akhter J., Chua T.C., Shehata M., Alzahrani N., Al-Alem I., Morris D.L. (2015). Heat sink effect on tumor ablation characteristics as observed in monopolar radiofrequency, bipolar radiofrequency, and microwave, using ex vivo calf liver model. Medicine.

[11] Fabi S.G. (2015). Noninvasive skin tightening: Focus on new ultrasound techniques. Clin. Cosmet. Investig. Dermatol..

[12] Sanghvi N. (2004). Non-invasive surgery using high intensity focused ultrasound (HIFU). Med. Phys..

[13] Shaw C.J., Rivens I., Civale J., Botting K.J., Giussani D.A., ter Haar G., Lees C.C. (2017). High Intensity Focused Ultrasound (HIFU): A method of non-invasive placental vascular occlusion. Bjog-Int. J. Obstet. Gy.

[14] Ko E.J., Hong J.Y., Kwon T.R., Choi E.J., Jang Y.J., Choi S.Y., Yoo K.H., Kim S.Y., Kim B.J. (2017). Efficacy and safety of non-invasive body tightening with high-intensity focused ultrasound (HIFU). Skin Res. Technol..

[15] Guan L.M., Xu G. (2016). Damage effect of high-intensity focused ultrasound on breast cancer tissues and their vascularities. World J. Surg. Oncol..

[16] Kennedy J.E., Ter Haar G.R., Cranston D. (2003). High intensity focused ultrasound: Surgery of the future?. Br. J. Radiol..

[17] Sung H.H., Jeong B.C., Seo S.I., Jeon S.S., Choi H.Y., Lee H.M. (2012). Seven years of experience with high-intensity focused ultrasound for prostate cancer: Advantages and limitations. Prostate.

[18] He P., Shou W., Duan S., Xia R. Dual-frequency High Intensity Focused Ultrasound (HIFU) Accelerating Therapy. Proceedings of the 2005 IEEE Engineering in Medicine and Biology 27th Annual Conference.

[19] Illing R.O., Kennedy J.E., Wu F., Ter Haar G.R., Protheroe A.S., Friend P.J., Gleeson F.V., Cranston D.W., Phillips R.R., Middleton M.R. (2005). The safety and feasibility of extracorporeal high-intensity focused ultrasound (HIFU) for the treatment of liver and kidney tumours in a Western population. Br. J. Cancer.

[20] Li S., Wu P.H. (2013). Magnetic resonance image-guided versus ultrasound-guided high-intensity focused ultrasound in the treatment of breast cancer. Chin. J. Cancer.

[21] Lee J.D., Huang C.H., Yang S.T., Chu Y.H., Shieh Y.Y., Chen J.W., Lin K.J. (2013). MRI/SPECT-based diagnosis and CT-guided high-intensity focused-ultrasound treatment system in MPTP mouse model of Parkinson’s disease. Med. Eng. Phys..

[22] Owen N.R., Chapelon J.Y., Bouchoux G., Berriet R., Fleury G., Lafon C. (2010). Dual-mode transducers for ultrasound imaging and thermal therapy. Ultrasonics.

[23] Hasebroock K.M., Serkova N.J. (2009). Toxicity of MRI and CT contrast agents. Expert Opin. Drug Metab. Toxicol..

[24] Ansari T., Yousef A., El Gamassy A., Fayez M. (2014). Ultrasound-guided spinal anaesthesia in obstetrics: Is there an advantage over the landmark technique in patients with easily palpable spines?. Int. J. Obstet. Anesth..

[25] Chan V.W.S., Perlas A., Rawson R., Odukoya O. (2003). Ultrasound-guided supraclavicular brachial plexus block. Anesth. Analg..

[26] Brenner K., Ergun A.S., Firouzi K., Rasmussen M.F., Stedman Q., Khuri-Yakub B.P. (2019). Advances in Capacitive Micromachined Ultrasonic Transducers. Micromachines.

[27] Sanghvi N.T., Chen W.H., Carlson R., Weis C., Seip R., Uchida T., Marberger M. (2017). Clinical validation of real-time tissue change monitoring during prostate tissue ablation with high intensity focused ultrasound. J. Ther. Ultrasound.

[28] Pak C.S., Lee Y.K., Jeong J.H., Kim J.H., Seo J.D., Heo C.Y. (2014). Safety and efficacy of ulthera in the rejuvenation of aging lower eyelids: A pivotal clinical trial. Aesthet. Plast Surg..

[29] Qi W.J., Li R., Ma T., Shung K.K., Zhou Q.F., Chen Z.P. (2014). Confocal acoustic radiation force optical coherence elastography using a ring ultrasonic transducer. Appl. Phys. Lett..

[30] Shin E.J., Kang B., Chang J.H. (2018). Real-Time HIFU Treatment Monitoring Using Pulse Inversion Ultrasonic Imaging. Appl. Sci..

[31] Mlosek R.K., Malinowska S., Sikora M., Debowska R., Stepien A., Czekaj K., Dabrowska A. (2013). The use of high frequency ultrasound imaging in skin moisturization measurement. Skin Res. Technol..

[32] Schuetzenberger K., Pfister M., Messner A., Froehlich V., Garhoefer G., Hohenadl C., Schmetterer L., Werkmeister R.M. (2019). Comparison of optical coherence tomography and high frequency ultrasound imaging in mice for the assessment of skin morphology and intradermal volumes. Sci. Rep..

[33] Saijo Y., Kobayashi K., Okada N., Hozumi N., Hagiwara Y., Tanaka A., Iwamoto T. High Frequency Ultrasound Imaging of Surface and Subsurface Structures of Fingerprints. Proceedings of the 2008 30th Annual International Conference of the IEEE Engineering in Medicine and Biology Society.

[34] Lim H.G., Li Y., Lin M.Y., Yoon C., Lee C., Jung H., Chow R.H., Shung K.K. (2016). Calibration of Trapping Force on Cell-Size Objects from Ultrahigh-Frequency Single-Beam Acoustic Tweezer. IEEE Trans. Ultrason. Ferroelectr. Freq. Control.

[35] Lim H.G., Shung K.K. (2017). Quantification of Inter-Erythrocyte Forces with Ultra-High Frequency (410 MHz) Single Beam Acoustic Tweezer. Ann. Biomed. Eng..

[36] Lim H.G., Kim H.H., Yoon C. (2018). Evaluation method for acoustic trapping performance by tracking motion of trapped microparticle. Jpn. J. Appl. Phys..

[37] Lim H.G., Liu H.C., Yoon C.W., Jung H., Kim M.G., Yoon C., Kim H.H., Shung K.K. (2020). Investigation of cell mechanics using single-beam acoustic tweezers as a versatile tool for the diagnosis and treatment of highly invasive breast cancer cell lines: An in vitro study. Microsyst. Nanoeng..

[38] Lim H.G., Lee O.J., Shung K.K., Kim J.T., Kim H.H. (2020). Classification of Breast Cancer Cells Using the Integration of High-Frequency Single-Beam Acoustic Tweezers and Convolutional Neural Networks. Cancers.

[39] Soneson J.E. A User-Friendly Software Package for HIFU Simulation. Proceedings of the 8th International Symposium on Therapeutic Ultrasound.

[40] Soneson J.E. (2017). Extending the Utility of the Parabolic Approximation in Medical Ultrasound Using Wide-Angle Diffraction Modeling. IEEE Trans. Ultrason. Ferroelectr. Freq. Control..

[41] Wang Z., Bai J., Li F., Du Y., Wen S., Hu K., Xu G., Ma P., Yin N., Chen W. (2003). Study of a "biological focal region" of high-intensity focused ultrasound. Ultrasound Med. Biol..

[42] Gill I.S., Hsu T.H., Fox R.L., Matamoros A., Miller C.D., Leveen R.F., Grune M.T., Sung G.T., Fidler M.E. (2000). Laparoscopic and percutaneous radiofrequency ablation of the kidney: Acute and chronic porcine study. Urology.

[43] Corwin T.S., Lindberg G., Traxer O., Gettman M.T., Smith T.G., Pearle M.S., Cadeddu J.A. (2001). Laparoscopic radiofrequency thermal ablation of renal tissue with and without hilar occlusion. J. Urol..

[44] Nakada S.Y., Jerde T.J., Warner T.F., Lee F.T. (2004). Comparison of radiofrequency ablation, cryoablation, and nephrectomy in treating implanted VX-2 carcinoma in rabbit kidneys. J. Endourol..

[45] Kornstein A.N. (2012). Ulthera for silicone lip correction. Plast Reconstr. Surg..

[46] Friedmann D.P., Fabi S.G., Goldman M.P. (2014). Combination of intense pulsed light, Sculptra, and Ultherapy for treatment of the aging face. J. Cosmet. Dermatol..

[47] Ma T., Yu M., Li J., Munding C.E., Chen Z., Fei C., Shung K.K., Zhou Q. (2015). Multi-frequency intravascular ultrasound (IVUS) imaging. IEEE Trans. Ultrason. Ferroelectr. Freq. Control..

[48] Munding C.E., Cherin E., Jourard I., Weyers J.J., Goertz D.E., Courtney B.K., Foster F.S. (2018). Development of a 3 French Dual-Frequency Intravascular Ultrasound Catheter. Ultrasound Med. Biol..

[49] Bove T., Zawada T., Serup J., Jessen A., Poli M. (2019). High-frequency (20-MHz) high-intensity focused ultrasound (HIFU) system for dermal intervention: Preclinical evaluation in skin equivalents. Skin Res. Technol..

[50] Xing Y., Lu X., Pua E.C., Zhong P. (2008). The effect of high intensity focused ultrasound treatment on metastases in a murine melanoma model. Biochem. Biophys. Res. Commun..

[51] Wang R.S., Liu L.X., Gu Y.H., Lin Q.F., Guo R.H., Shu Y.Q. (2010). The effect of endostatin and gemcitabine combined with HIFU on the animal xenograft model of human pancreatic cancer. Biomed. Pharmacother..

