# Supplementary material for: Thermal Ablation and High-Resolution Imaging Using a Back-to-Back (BTB) Dual-Mode Ultrasonic Transducer: In Vivo Results
Source: Sensors (Basel). 2021 Feb 24;21(5):1580. doi: 10.3390/s21051580 (PMC7956793; doi:10.3390/s21051580)
Supplement: Supplementary file 1 [file sensors-21-01580-s001.zip › Supplementary Materials.docx]

Supplementary Materials

Article

Thermal Ablation and High-Resolution Imaging using a Back-to-back (BTB) Dual-Mode Ultrasonic Transducer: *In vivo* Results

Hae Gyun Lim^1, †^, Hyunhee Kim^2, †^, Kyungmin Kim^1^, Jeongwoo Park^2^, Yeonggeun Kim^1^, Jinhee Yoo^2^, Dasom Heo^3^, Jinhwan Baik^1^, Sung-Min Park^1^, and Hyung Ham Kim^1, *^

^1^Department of Creative IT Engineering, Pohang University of Science and Technology, Pohang 37673, Republic of Korea; [haegyun@postech.ac.kr](mailto:haegyun@postech.ac.kr) (H.G.L.); [kyungmin10@postech.ac.kr(K.K.)](mailto:kyungmin10@postech.ac.kr(K.K.)); [gun9509@postech.ac.kr](mailto:gun9509@postech.ac.kr) (Y.K.); [jinhwan52@postech.ac.kr](mailto:jinhwan52@postech.ac.kr) (J.B.); [sungminpark@postech.ac.kr](mailto:sungminpark@postech.ac.kr) (S.M.P.); [david.kim@postech.ac.kr](mailto:david.kim@postech.ac.kr) (H.H.K.)

^2^School of Interdisciplinary Bioscience and Bioengineering, Pohang University of Science and Technology, Pohang 37673, Republic of Korea; [rlagusgml036@postech.ac.kr](mailto:rlagusgml036@postech.ac.kr) (H.K); [jpark215@postech.ac.kr](mailto:jpark215@postech.ac.kr) (J.P.); [jinhee.yoo@postech.ac.kr](mailto:jinhee.yoo@postech.ac.kr) (J.Y.);

^3^Department of Mechanical Engineering, Pohang University of Science and Technology, Pohang 37673, Republic of Korea; [hds9385@postech.ac.kr](mailto:hds9385@postech.ac.kr) (D.H.)

* Correspondence: [david.kim@postech.ac.kr](mailto:david.kim@postech.ac.kr)

^†^ These authors contributed equally to this work.


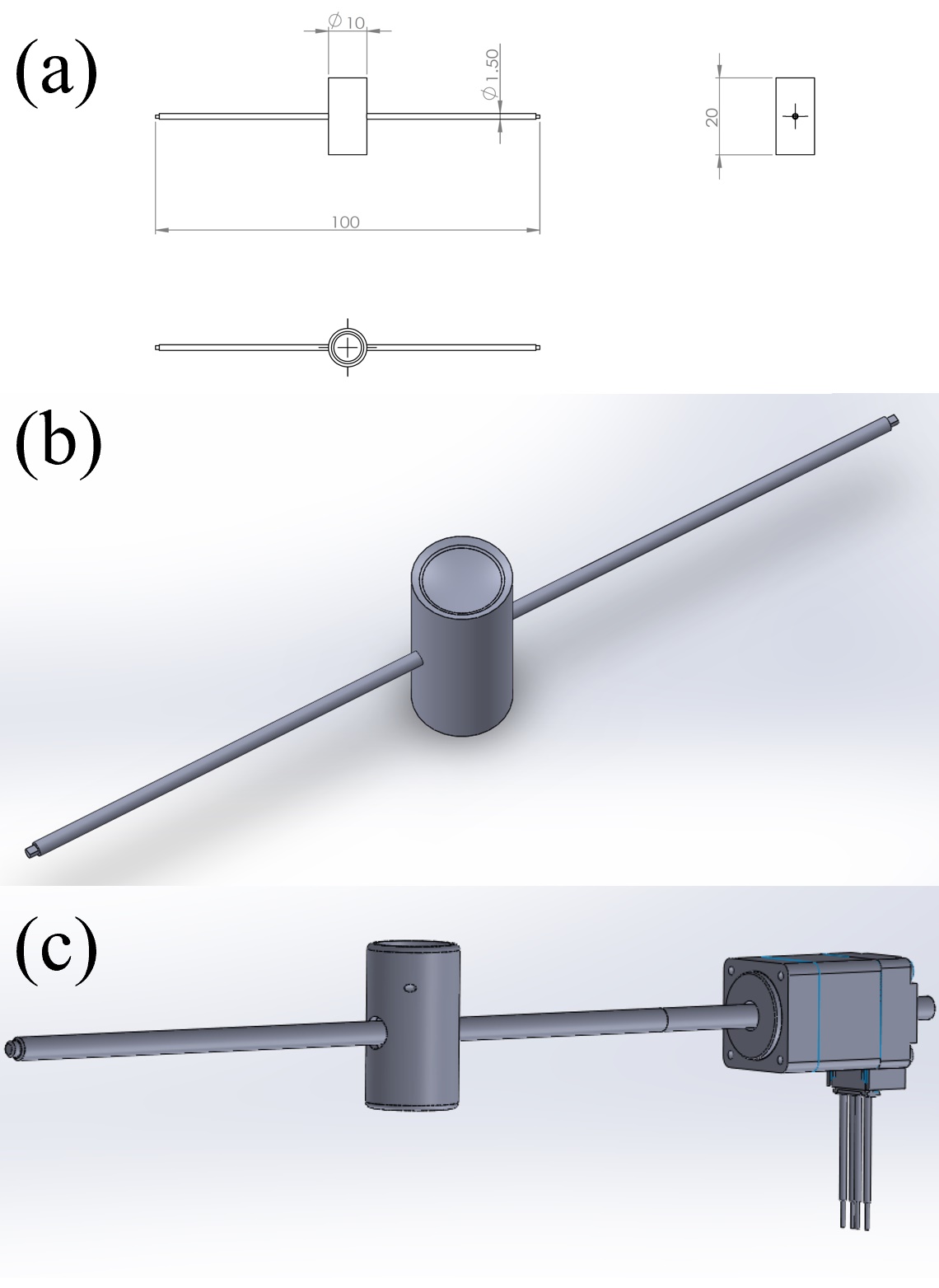


**Figure S1.** Computer-aided design (Solidworks, Dassault Systèmes Solidworks Corporation, MA, USA) for the back-to-back (BTB) transducer and the stepper motor for the rotation (PKP213D05A, Oriental Motor, Tokyo, Japan)


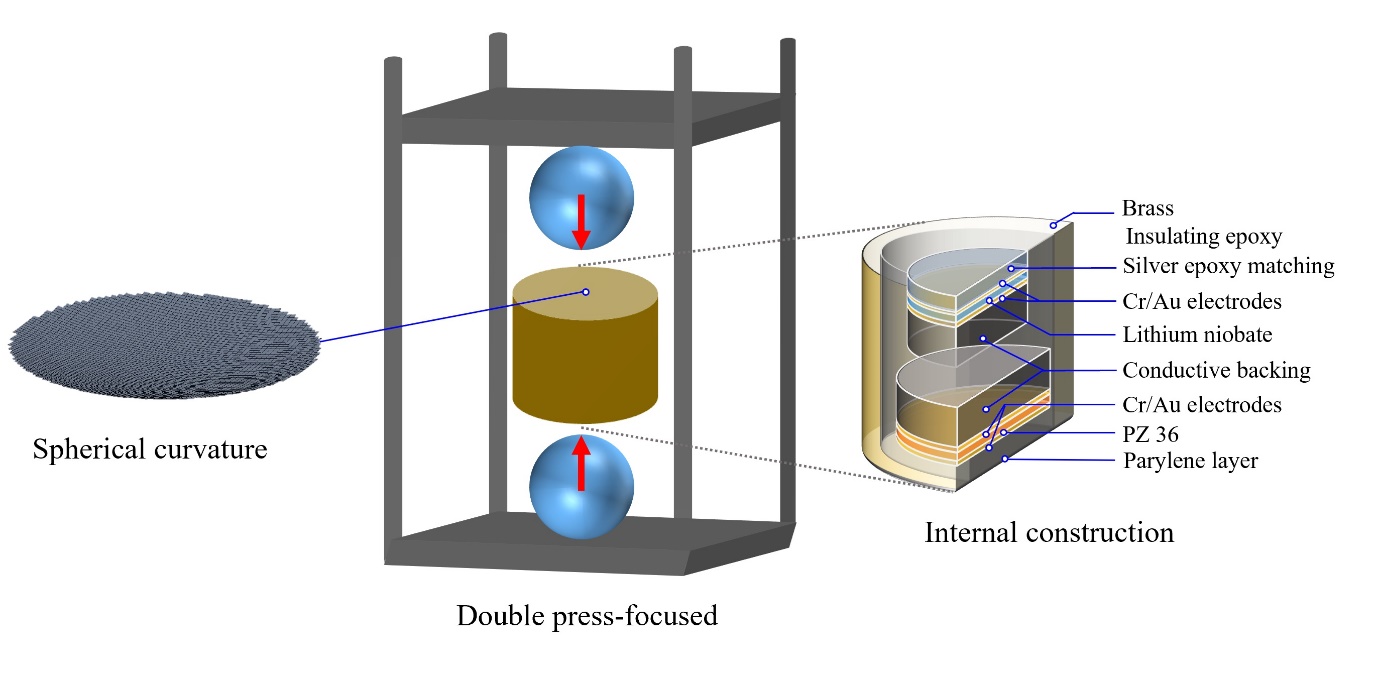


**Figure S2.** Schematics of a press-focusing method for the BTB transducer


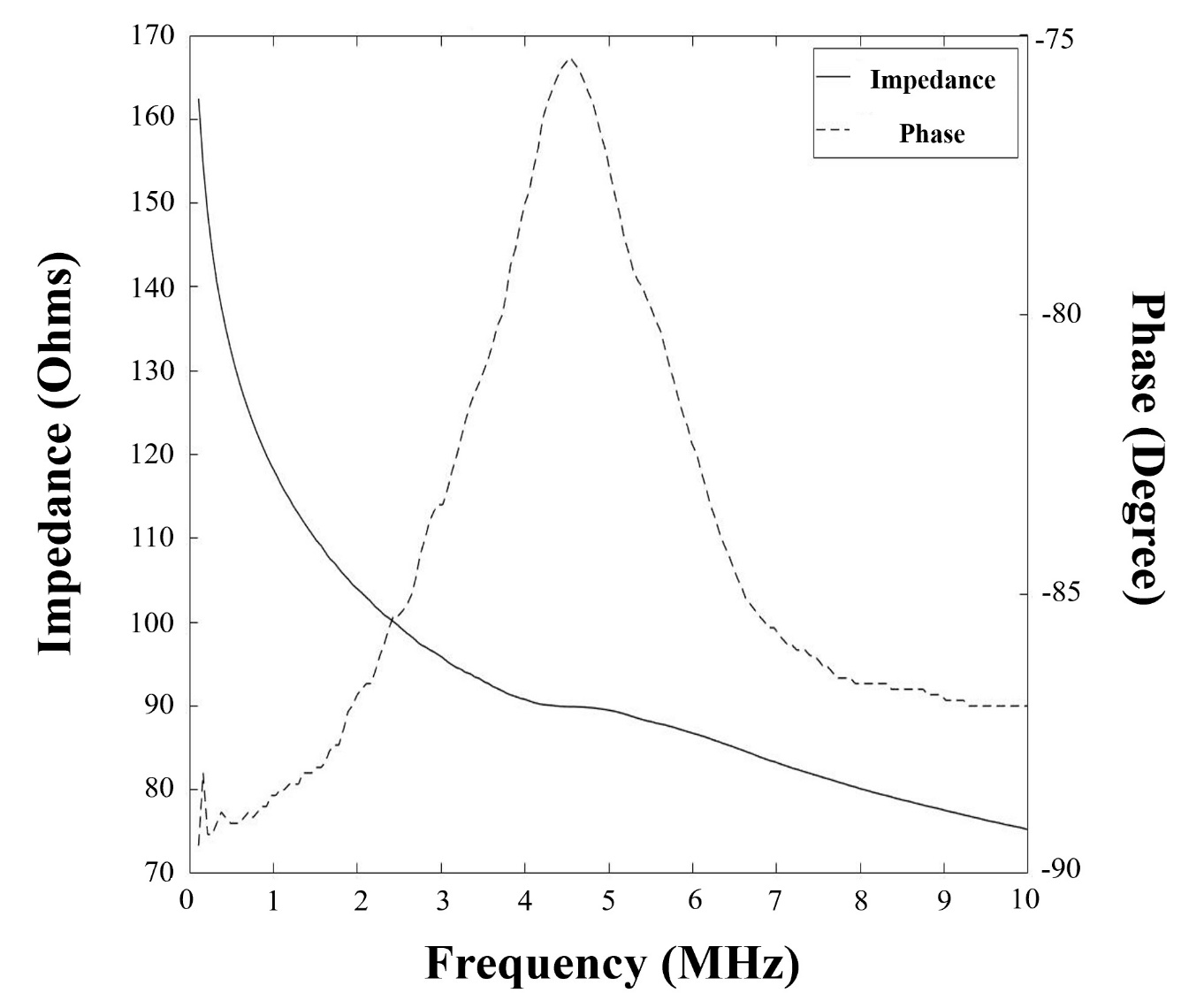


**Figure S3.** The electrical impedance and phase of the HIFU transducer measured by impedance analyzer (E4990A, Keysight Technologies, Santa Rosa, CA, USA)

**Video S1.** 2D B-scan frame collection. B-scan ultrasound images before and after treatment with arrows showing liver and small intestine. Rectangular box represents the thermal ablation region due to HIFU. Total duration: 28 seconds. Frame rate: 20 f/s

**Video S2.** 2D C-scan frame collection. C-scan ultrasound images before and after treatment with arrows showing bone (blue color) and liver (yellow and red color). Because of a strong ultrasound reflection from bone, data below bone was only acquired for imaging. Rectangular box represents the thermal ablation region generated after HIFU. Total duration: 27 seconds. Frame rate: 20 f/s
